# Adapting the WEPP Hillslope Model and the TLS Technology to Predict Unpaved Road Soil Erosion

**DOI:** 10.3390/ijerph19159213

**Published:** 2022-07-28

**Authors:** Yi Wang, Wei He, Ting Zhang, Yani Zhang, Longxi Cao

**Affiliations:** 1 Key Laboratory of Ministry of Education on Land Resources Evaluation and Monitoring in Southwest China, Sichuan Normal University, Chengdu 610066, China; wy@sicnu.edu.cn; 2 Institute of Geography and Resources Science, Sichuan Normal University, Chengdu 610101, China; hewei5196@163.com; 3 College of Ecology and Environment, Chengdu University of Technology, Chengdu 610059, China; ztcdut2022@163.com (T.Z.); zyn2389084945@163.com (Y.Z.)

**Keywords:** road erosion, erosion modelling, WEPP, terrestrial laser scanning, South China

## Abstract

Unpaved road erosion have been recognized as important sediment sources in a watershed. To evaluate where and when road erosion occurs, the soil loss along road segments should be precisely predicted with process-based erosion models. Methods: The hillslope version of the Water Erosion Prediction Project (WEPP) was used to estimate soil loss from 20 typical road segments in the red soil region of South China. Terrestrial laser scanning (TLS)-measured soil losses were used to validate the model simulations. The results showed that the WEPP model could reasonably predict the total soil loss in relatively short (less than 100 m) and gentle (slope gradient lower than 10%) road segments. In contrast, soil loss would be underestimated for long or steep road segments. Detailed outputs along roads revealed that most of the peak soil loss rates were underestimated. It might due to the linear critical shear stress theory in the WEPP model. Additionally, the lack of upstream flow was found to be connected to the relatively low model efficiency. Nevertheless, the WEPP simulation could accurately fit erosion trend and predict the peak soil loss positions along road segments. Conclusions: The WEPP model could be adopted to evaluate the erosion risk of unpaved roads in the red soil region of South China.

## 1. Introduction

Unpaved roads are common man-made geographical features that are distributed in agricultural or forested watersheds [1]. Road construction will change the underlying topography and alter the surface hydrology [2]. In turn, runoff generation is enhanced and results in a higher soil loss risk than that in other land-use types, such as farmland [3,4]. Furthermore, roads may accumulate, deliver, or increase sediment in runoff [5,6]. Therefore, unpaved roads have been reported to contribute a large proportion of sediment yield in a watershed, although they generally occupy a small fraction of the area [3,6,7,8,9]. Road-related sediment that is routed to water bodies such as lakes and rivers can cause serious damage to the aquatic environment and impair the beneficial uses of surface water resources [10]. Thus, road erosion should be considered one of the main sediment sources and should be properly evaluated [11,12,13,14].

Soil erosion models are effective tools for road erosion evaluation [15]. During the last 20 years, different models have been applied and developed in road-related soil loss prediction. For example, the USLE (Universal Soil Loss Equation) [16] and its modifications have been used to estimate sediment production from forest roads [17]. Scientists in North America have developed several empirical models to predict annual road sediment; examples include the SEDMODL (https://www.ncasi.org/resource/sedmodl-2-0/, accessed on 30 June 2022) [18], WARSEM [19], ROADMOD [20] and READI [13]. Some of these models have also been applied in other regions, such as Australia [21], China [22], Iran [23] and Turkey [24]. However, as empirical road erosion models are mainly based on research in local regions, they are difficult to apply to areas with different environmental characteristics. Furthermore, most of the abovementioned empirical models can evaluate only the total annual road soil loss.

Process erosion models are widely used due to their inherent physically based capabilities for estimating the spatial and temporal distributions of soil losses [25]. The Water Erosion Prediction Project (WEPP) is a physically based, distributed model that simulates the detachment, transport and deposition of sediment by surface runoff [26]. It is one of the most popular process-based models that has been adopted around the world [27,28,29,30,31]. Elliot et al., (1995) proved that the WEPP hillslope version could be an efficient tool for estimating soil loss from unpaved forest roads [32]. Based on a database of road-related soil and terrain files, the US Forest Service has developed a specific interface named WEPP:Road (https://forest.moscowfsl.wsu.edu/fswepp/, accessed on 30 June 2022) for forest road erosion estimation [33]. This web-based interface provided an efficient way to model soil erosion on individual road segments. With the help of GPS and GIS technologies, the WEPP:Road could be adopted to predict soil loss from road networks at the watershed scale (with an area of 3040 km^2^) [34]. The hillslope version of the WEPP has also been used to predict sediment yield from road segments. For example, Brown et al., (2013) simulated soil loss from forest roads with the WEPP model and evaluated the effect of management practices in reducing sediment delivery to streams [35]. Guerra and Silva (2011) adopted the WEPP model to predict sediment generation from 4 road segments [36]. In most of the above studies, the WEPP model was used to evaluate the total soil loss from road segments and then validated with field sediment yield data from sediment traps. It should be noted that the WEPP is a process-based model and can simulate the detailed soil loss spatially, specifically along a hillslope. This information is of vital importance for road erosion risk evaluation and precise conservation. However, it is difficult to validate the detailed model results along a road segment using only the total soil loss information [25].

Terrestrial laser scanning (TLS) technology can generate high-resolution micro-terrain data based on a 3D point cloud. With the help of TLS technology, scientists can precisely measure the detailed erosion volume and morphology information along eroded rills [37,38]. Cao et al., (2021) quantified rill erosion with high precision (grid resolution of 5 cm) along road segments and explored the influential factor with TLS technology [4]. The results verified the reliability of adopting TLS technology for erosion measurement on smooth road surfaces. Therefore, the measured erosion information should be helpful in validating the WEPP-simulated soil loss along road segments. This study was conducted based on our previous TLS-aided road erosion measurements. Soil losses from road segments were simulated by the WEPP hillslope version. The objectives of this study were to (1) validate the precision of the WEPP model in predicting soil loss along road segments of different lengths and slope gradients, and (2) explore the unique factors that determine the model efficiency in unpaved road conditions. The results are beneficial for enlarging the scope of application for the WEPP model, and they are also helpful for road erosion risk evaluation and conservation.

## 2. Materials and Methods

### 2.1. The Study Site

**Figure 1:**
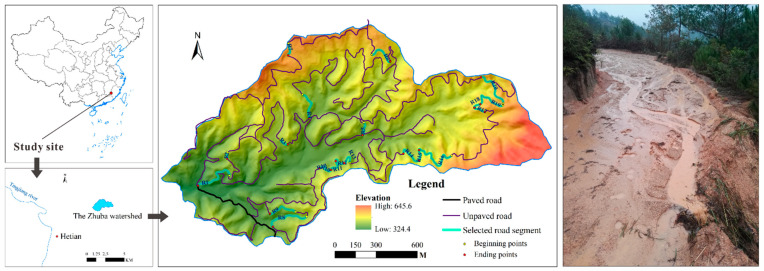
The location of the study site and a typical road segment where rill erosion occurring.

The Zhuba watershed in Fujian Province of Southeast China (116°00′45″~116°39′20″ E, 25°18′40″~26°02′05″ N) was chosen as the study site (Figure 1). It is a typical hilly small watershed with an area of 2.32 km^2^. The study site is dominated by a wet subtropical monsoon climate with an average annual precipitation of 1795 mm, among which approximately 75.7% occurs in the wet season (from March to August). The elevation in the watershed ranges from 324 m to 646 m above sea level, and the average slope is 23 degrees. The local soil is derived from granitic weathered materials and is characterized by a coarse texture, low organic matter and high erodibility [39]. The study site is a forested watershed, and approximately 50% has been opened for *Camellia oleifera* planting. An unpaved road network with a total length of 23.97 km was built in 2011. The road widths range from 3 m to 5 m to allow small vehicles to pass through for *C. oleifera* maintenance. Due to the low engineering standards, most of the roads were built by simply removing the topsoil downhill with a crawler tractor without erosion conservation measures. Serious rill erosion along roads and gullies can be found in some cases (Figure 1). To ensure that road surfaces are passable for vehicles, the road surface should be maintained, and rills should be filled or cleaned after the wet season. More details about the road network and soil loss can be found in Cao et al., (2021) [4].

### 2.2. TLS-Aided Road Erosion Measurement

Before the TLS erosion measurement, 20 road segments were chosen throughout the watershed by field survey in November 2018. These road segments were selected according to slope gradients, lengths and surface erosion conditions to ensure they were representative of the road network at the study site. Each road segment could be treated as a hillslope starting from the high point to the adjacent low ending point. One road segment is hydrologically independent from other road segments and therefore contains an entire process of erosion initiation, intensification and general deposition at the ending point [4]. The TLS measurement was conducted with a FARO Focus S150 3D Laser Scanner. The scanning speed was 97,600 points per second with a point cloud density of 1.5 mm at a 10 m distance. In most cases, road surface rill micro-terrain was measured using a station scanning manner along roads with average intervals of approximately 10 m. More than one scan would be performed at a site if the site had intensive rill erosion or complicated terrain conditions. With the help of CloudCompareV2.9.1, the original point cloud data could be registered and then converted to a 0.05 m resolution raster digital elevation model (DEM). According to the DEM data, road surface rills could be extracted, and erosion amounts could be calculated with the Spatial Analyst Tools in the ESRI ArcGIS 10.3 environment. Detailed information on the measured road segments and the process for road surface rill extraction has been demonstrated by Cao et al., (2021) [4]. Table 1 lists the TLS-measured road segment erosion and terrain factors.

### 2.3. Road Erosion Modelling

The WEPP hillslope version 2012.8 was used to predict soil loss from road segments in this study. The model simulates water and sediment detachment and transport through a series of overland flow elements (OFEs) [34]. According to the field survey, road surface rill erosion is the dominant erosion form throughout the road networks in the watershed. Therefore, the road surface was simulated as an OFE for each segment. There are four input files required to run the hillslope WEPP model: (1) the climate file describing the temporal variability of weather data; (2) the soil file containing the textural and hydraulic properties of the soils in each OFE; (3) the slope file including the steepness along the hillslope profile; and (4) the plant/management lists of the specific management scenarios and plant growth properties for each OFE. Considering the WEPP:Road model has provided detailed parameters for WEPP application and has been proved to be reliable by scientists [33,34]. This study would run the WEPP model according to the input combinations of WEPP:Road.

#### 2.3.1. The Climate File

**Figure 2:**
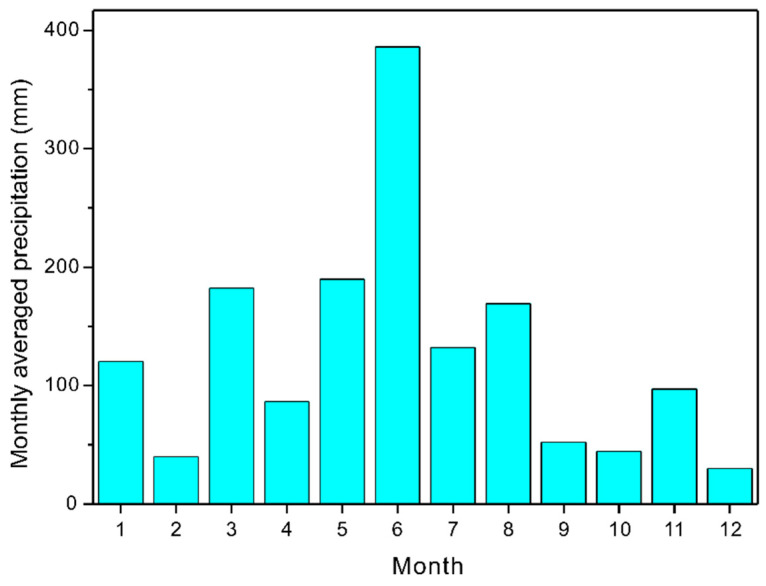
The monthly averaged precipitation of 2017 and 2018 at the study site.

The climate data used in this study were recorded by the Hetian Soil and Water Conservation Monitoring Station, which is approximately 6 km from the Zhuba watershed. Since the TLS measurement was conducted in late 2018 and the roads were maintained in last November, the weather data for both 2017 and 2018 were considered (Figure 2). The monthly averaged precipitation, wet days, maximum temperature, and minimum temperature for the two years were collected to create a custom PAR file with the Rocky Mountain Research Station Climate Generator (https://forest.moscowfsl.wsu.edu/cgi-bin/fswepp/wr/wepproad.pl, accessed on 30 June 2022). In turn, the PAR file was uploaded to CLIMGEN [40] in WEPP Windows to create a 2-year daily average climate file.

#### 2.3.2. The Soil File

The local soils are derived from a layer of highly erodible sandy granite weathered material (Grus) with a thickness of approximately 20 m [39]. Due to road construction disturbance, the original topsoil layers were removed, and road surfaces were composed of coarse granite materials. Surface soil samples were taken from each road segment to determine property indexes. The soil texture in the study area is sandy loam with average contents of 65.24% sand, 19.02% silt and 15.74% clay. Soil organic matter was measured to range from 2.64 to 5.97 g/kg with an average value of 4.33 g/kg. The averaged cation exchange capacity (CEC) was measured as 4.67 meq/100 g. The bulk density was measured as range from 1.39 to 1.60 g/cm^3^ with an average value of 1.49 g/cm^3^.

The rill erodibility, critical shear stress, interrill erodibility and effective hydraulic conductivity are the most sensitive parameters for WEPP modelling. Elliot and Hall (2010) provided a parameter database according to soil texture, road design and road traffic level [15]. We cited this database in the WEPP:Road interface by selecting the suitable combination of road design, soil texture and traffic levels. According to the characteristics of the studied road network, the road surface soil texture was selected as sandy loam, road design was selected as insloped bare ditch, road surface was selected as native and traffic level was low. Soil parameters information could be presented by checking the Soil Texture button in the interface. The soil parameters used for WEPP modelling in this study are listed in Table 2.

#### 2.3.3. The Terrain File

The terrain files were created according to the detailed topography information measured by TLS technology. First, the elevation profile was extracted from the 0.05 m-resolution TLS DEM with the central line of each road segment. In turn, the road segment was divided into subsegments according to the elevation profile variation along the road. The subsegments were characterized as having a homogeneous slope gradient, and the length ranged from 10 m to 50 m, which was determined by both the gradient variation and the total road length. After road segmentation, the slope gradient could be calculated for each subsegment by dividing the length by the elevation difference. Then, both the slope gradient and the length data of subsegments were inputted to the WEPP Slope Profile Editor, and finally, a terrain file was created for a road segment.

#### 2.3.4. The Management Input

For security reasons, most of the roads in the study site were built as insloped surfaces. As a result, rill erosion develops along the inner side of roads. According to the field survey, there is little vegetation on the road surface and eroded rills. Therefore, the management layer input was selected as road-insloped with bare ditches in the management database.

### 2.4. Data Analysis

The Nash-Sutcliffe model efficiencies (MEs) [41] were used to evaluate the precision of the modelling results according to the following equation:

(1) $$ME=1-\frac{\sum {\left(Q_{i}-Q_{c}\right)}^{2}}{\sum {\left(Q_{i}-Q_{m}\right)}^{2}}$$

where *ME* is the model efficiency; *Q_i_* is the measured soil loss value; *Q_c_* is the WEPP predicted soil loss value; and *Q_m_* is the average of the measured soil loss values. The performance of *ME* is given as follows: *ME* > 0.75 (very good), 0.75 ≥ *ME* > 0.65 (good), 0.65 ≥ *ME* > 0.5 (satisfactory), and 0.5 ≥ (unsatisfactory) [42]. A negative *ME* means that the average of the measured values can better estimate the slope length than the model predicted values.

The effects of road segment terrain indexes on model efficiency were also evaluated by statistically analyzing the data. The Pearson correlation coefficient (R) was used to evaluate the relationship between model deviation and terrain factors along road segments. Regression analyses were conducted to establish equations between the measured and predicted values. The coefficient of determination (R^2^) was used to evaluate the equation performance. The results were reported at the α = 0.05 level of significance. All analyses were carried out with the SPSS 20.0 software package for Windows (IBM Corp., Armonk, NY, USA), while the graphics were generated with the ORIGIN 8.0 software package (OriginLab, Northampton, MA, USA).

## 3. Results

### 3.1. Predicted Total Soil Loss from Road Segments

**Figure 3:**
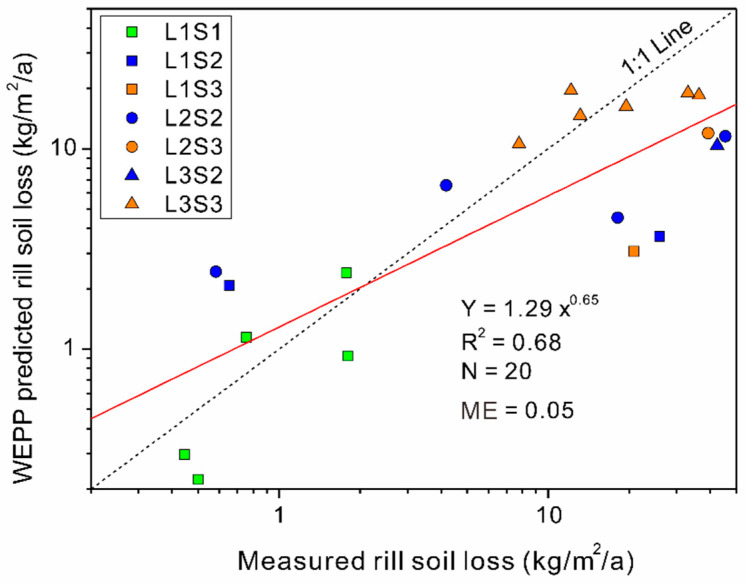
The relationship between the TLS-measured and WEPP-predicted rill soil loss values. L1, L2 and L3 represent road segment lengths shorter than 100 m, 100 m–200 m and longer than 200 m, respectively. S1, S2 and S3 represent road gradients lower than 5%, 5–10% and higher than 10%, respectively.

Considering that the unpaved roads in the study site were maintained annually and the road surface rills were cleaned after the wet season, the measured soil loss rate could be treated as the annual erosion rate. Therefore, the TLS-measured rill soil loss rates were comparable to the WEPP-predicted soil loss. Figure 3 illustrates the WEPP-predicted rill soil loss rate for each road segment plotted with the measured values. The 20 road segments were classified into 7 groups according to different lengths and gradients. For the total dataset, the low *ME* value of 0.05 implies an unsatisfactory WEPP prediction. The model variation ratio (the predicted rill soil loss/the measured rill soil loss) was ranged from 0.18 to 5.68 with the averaged value of 1.43. This result is mainly due to the underestimated rill soil loss rates in the case of high erosion risks. A power function with an exponent of 0.65 could be used to describe the relationship between the measured and predicted soil loss rates. Further comparison was made under different road terrain conditions. In the case of road gradients lower than 5%, all road lengths were shorter than 100 m, and the WEPP-predicted soil losses were generally close to the 1:1 line (the green rectangle in Figure 3). When the road gradients were within the range from 5% to 10% (the blue points in Figure 3), the WEPP prediction would overestimate the soil losses, which were lower than 10 kg/m^2^. For the measured soil loss higher than 10 kg/m^2^, the WEPP model would result in underestimation. In the case of road gradients steeper than 10% (the orange points in Figure 3), the soil losses were underestimated for 5 of 8 road segments. For the different lengths, the soil loss from most of the road segments shorter than 100 m was overestimated by the WEPP except for two segments with relatively high gradients (the rectangular points in Figure 3). In contrast, the majority of WEPP-predicted soil loss rates were underestimated for road segments longer than 100 m (the circle and triangle points in Figure 3).

### 3.2. Predicted Detailed Soil Loss along Road Segments

**Figure 4:**
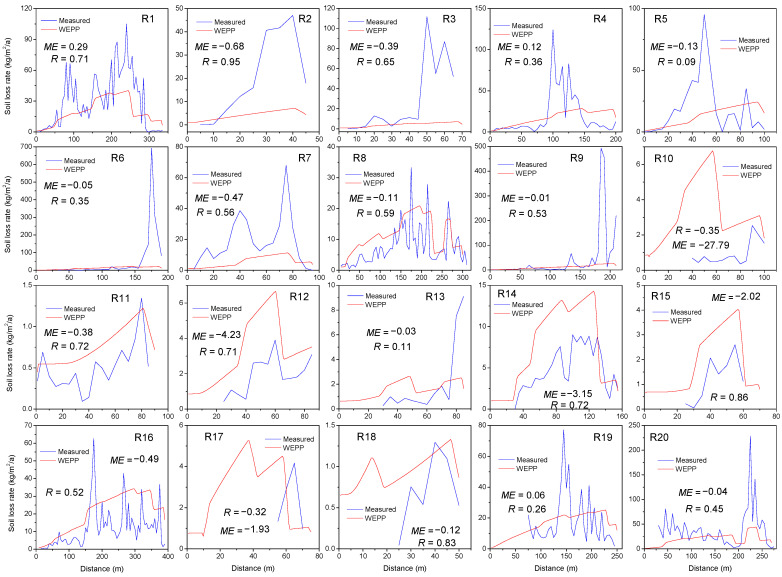
The TLS-measured and WEPP-predicted detailed soil loss along road segments.

The WEPP-predicted soil loss rates along road segments were extracted according to the Plot Output data and illustrated with the TLS-measured values, as shown in Figure 4. The predicted WEPP values generally underestimated the peak soil loss rates for road segments longer than 100 m, especially for some road segments with intensive soil loss and gully formation (R6, R9 and R20). On the other hand, the predicted soil loss rates were overestimated for many road segments shorter than 100 m (e.g., R10, R12, R15, R17 and R18). The ME values in Figure 4 show that the WEPP simulation provided low efficiency compared with the measured soil loss along roads. Nevertheless, it is important to note that the WEPP-predicted soil loss rate followed a similar trend to that of the measured values. This could be supported by the Pearson correlation coefficient (R) for most road segments. As illustrated in Figure 4, the ascending or descending sections of the WEPP simulation were generally consistent with the measured soil loss rate along most road segments. Further regression analyses were conducted to quantify the relationship between the predicted and measured soil losses. Table 3 lists the regression equations for each road segment. The measured soil loss could be expressed as linear or power functions of the predicted values. For most of the road segments, the R^2^ values were significant at the 0.05 level.

**Figure 5:**
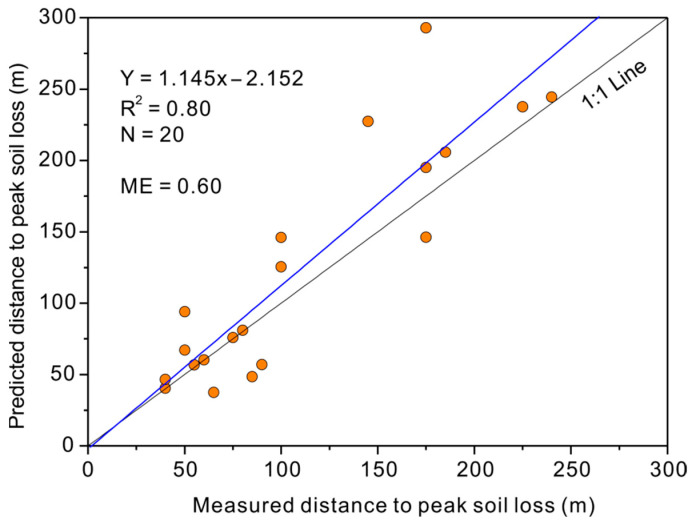
The measured distance to the peak soil loss as plotted with the predicted values.

A consistent trend could also be found from the peak values. Although the simulated values generally underestimated the peak soil loss rate in most cases, the positions of the peak soil loss along road segments could be simulated by the WEPP. As illustrated in Figure 4, the positions of the WEPP-predicted and TLS-measured maximum soil loss rates were generally close to each other. Further analysis was performed by plotting the measured distance from the road top to the maximum peak soil loss rates with the predicted distances, as shown in Figure 5. The model efficiency of 0.6 illustrated satisfactory agreement between the predicted and measured values. It should be noted that the predicted distance was overestimated when the distance exceeded 100 m, implying a delayed simulation of peak soil loss along road segments.

### 3.3. Factors That Influencing Model Performance

To explore the efficiency of the predicted soil loss, the model deviations were calculated as the WEPP-predicted soil loss rates minus the TLS-measured values of the 20 road segments and then correlated with influential factors (Table 4). The results illustrated that the slope gradient and upstream flow area showed only slightly negative correlation with model deviations. On the other hand, the road tortuosity showed slightly positive correlation. Among all the factors, the upstream flow area per road length was negatively related with model deviations at the α = 0.05 level of significance. A step regression analysis was conducted to quantify the relationship between the upstream flow area per road length and model deviation:

D = −0.270UA − 0.821, R^2^ = 0.256, N = 20, *p* < 0.05 (2)

where D is the deviation of predicted road soil loss rate (kg/m^2^/a), UA is the upstream flow area per road length (m^2^/m).

The detailed WEPP-predicted deviations were further calculated and correlated with distance to road top and slope gradient along each road segment. Table 5 illustrates that many of the correlation coefficients (R values) did not show significance at the 0.05 level due to the complexity of model deviations along road segments. However, most of the deviations were negatively related to the distance to the road top. This result implies that the underestimations of the WEPP output likely increase with distance to the road top. However, the R values between the deviation and slope gradient were more positive than the negative relationship, which means that the underestimation of WEPP tended to be reduced in the case of a steep gradient.

## 4. Discussion

### 4.1. The Efficiency of WEPP for Total Road Segment Soil Loss Prediction

As illustrated in Figure 3, the WEPP model generally provided a reasonable simulation of total soil loss rates for road segments with relatively low gradients and short lengths. This result implies that the unpaved road surface follows the opinion that the spatial scale for hillslope profile simulations is typically <100 m in length [43]. On the other hand, the predicted values lead to underestimation in the case of relatively high total soil losses occurring on steep and long road segments. This result implies the potential inability of the WEPP model to simulate the high-risk soil losses from long road segments. It is consistent with Elliot et al., (1991) reported that WEPP shear detachment might cause underestimation at higher erosion rates [44]. On the contrary, Zheng et al., (2020) reported that the WEPP simulated average annual soil loss was overly responsive to slope length increases at steep slopes in croplands of Loess Plateau [31]. This difference might due to the relatively short slope length of their field plots (less than 40 m) as comparing with the road segment length in this study. Furthermore, road segment length is not only a simple slope length factor but also determining the road-intercepted upstream flow area [4].

**Figure 6:**
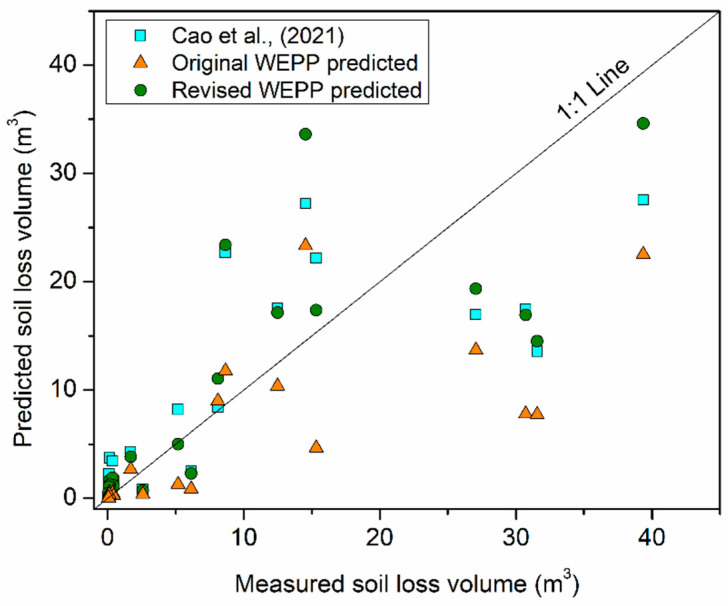
The efficiency of different predicted total road surface soil loss volumes. It could be seen that the revised WEPP predicted values are closer to the 1:1 line than the original WEPP values.

The hillslope version of the WEPP applied in this study treated each road segment as an independent hillslope and calculated road surface soil loss according to terrain factors, including slope length and gradient. As a linear feature, the importance of road-intercepted upstream flow area in predicting road soil loss has been verified by Cao et al., (2021) [4]. An empirical model (V = 0.205LS + 15.804A − 0.626) was built to predict erosion volumes according to road segment length (L), slope gradient (S) and intercepted upstream flow area (A). The WEPP-predicted soil loss amounts in this study were converted into erosion volumes and compared with those calculated using the equation developed by reference [4]. Figure 6 illustrates that the WEPP-predicted erosion volumes were generally lower than those calculated by the equation developed by reference [4]. The model efficiency for the WEPP erosion volume prediction was calculated as 0.38, lower than that from the equation reported by reference [4], which was 0.58. This result implies that the lack of road-intercepted upstream area might be another reason that leads to the underestimation of the hillslope version of the WEPP model. It also could be verified by the significant negative correlation between model deviation and upstream flow area per road length in Table 4. According to Equation (2), the model deviation could be estimated and then be used to revise the original WEPP predicted soil loss. Figure 6 showed that the revised WEPP predicted soil loss volumes were generally higher than the original values and closer to the 1:1 line. The model efficiency for the revised values was calculated as 0.59, which implies a satisfactory evaluation of road surface soil loss volume.

### 4.2. The Efficiency of WEPP for Detailed Soil Loss Prediction along Roads

Figure 4 shows that the WEPP-predicted detailed soil loss on short- or low-slope roads was relatively higher than the TLS-measured values. This result might be due to the interrill information in the detailed soil losses derived from the WEPP Plot Output data. For most parts of the road segments in Figure 4, the predicted detailed soil losses were lower than the measured values. This result could be explained by the fact that the soil detachment process in the WEPP model is simulated by the linearly critical shear stress model [45]. The flow shear stress on a straight hillslope would be calculated as linearly increasing with slope length or gradient [46]. This means that the simulated soil loss should also linearly increase with slope length, similar to the detailed simulated soil losses along road segments illustrated by Figure 4. Nevertheless, Cao et al., (2011b) reported that soil detachment should be predicted as a power function rather than a linear function on unpaved road surfaces [47]. This might explain the underestimation of the WEPP results on steep (>10%) and long (>100 m) road segments, especially for the peak soil loss, which was generally underestimated in this study. Meanwhile, the sediment equilibrium theory, which addresses sediment detachment and transportation by overland flow, may be another reason for the WEPP underestimation. With increasing slope length, more sediment would be transported, and in turn, the calculated detachment rate would be reduced [44]. This might explain the negative relationship between model deviation and slope length shown in Table 4, which implies increasing underestimations of WEPP output with distance to the road top. However, a steeper slope gradient along the hillslope would result in a higher soil detachment rate simulation and therefore could reduce the underestimation of the WEPP compared with the measured soil losses. The slope gradient variation and its effect on WEPP soil loss prediction are also supported by Zhang (2017), who found that an increased slope leads to a maximum soil loss along hillslopes [26].

Although the WEPP model underestimated soil loss from steep and long road segments, the predicted values followed a similar trend to the TLS-measured values. The significant fitness of the regression equations in Table 3 verified the high consistency between the WEPP-simulated and TLS-measured soil losses. The model efficiency calculated in Figure 5 illustrates that the peak soil loss positions could be reasonably estimated by the WEPP model. This verified the capability of the WEPP model in describing road slope variation and quantifying the response of road surface erosion. Additionally, road terrain factors would influence the abovementioned consistency.

The correlation coefficients which quantifying the match between measured and predicted erosion values in Figure 4 were relatively low for some long and steep road segments (e.g., R4, R5, R19 and R 20). This result supported the abovementioned effect of slope gradient and length in determining flow energy dynamics along hillslopes. Zhang (2017) reported that the WEPP model was extremely sensitive to slope steepness at longer slope lengths [26]. Therefore, a longer road length or steeper gradient might result in a larger deviation between the WEPP-predicted and the TLS-measured soil losses in this study. Additionally, as a linear feature, road tortuosity might play a role in determining the efficiency of the model results. The low correlation coefficient in some large road tortuosity (e.g., R13, R19) illustrates that the highly curved road segments tend to lead to a large deviation between the WEPP-predicted and TLS-measured soil loss rates. This result could be explained by the fact that the WEPP hillslope version treats road segments as straight slopes. However, the curved road segment implies a large variation in flow energy and sediment transport along the road surface compared with a straight hillslope. Therefore, these conditions might result in different soil loss processes along road segments than the WEPP simulations. The slightly active correlation between model deviation and road tortuosity in Table 4 implies that soil loss from some highly curved road segments might be over-estimated if they were modelled as straight slopes.

**Figure 7:**
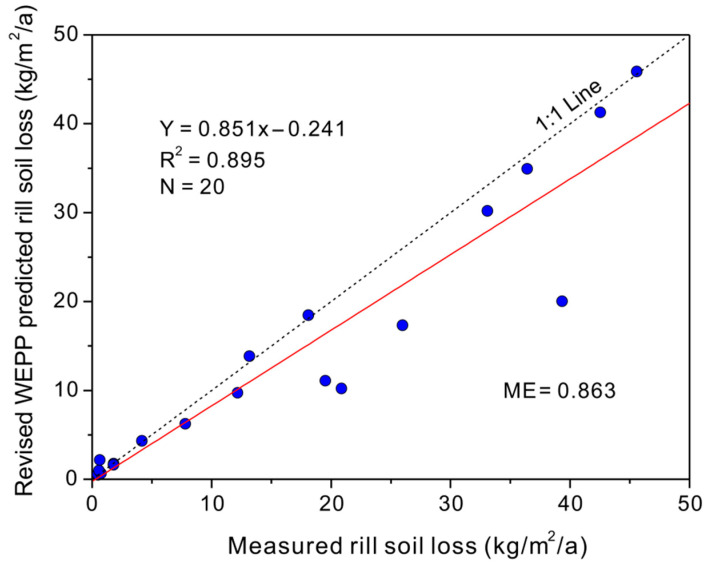
The measured soil loss plotted with the revised WEPP-predicted soil loss rates.

The functions developed in Table 3 could be applied to revise the WEPP-predicted total soil losses for each road segment. Figure 7 showed the revised model values plotted with the TLS-measured values. The revised WEPP-predicted total soil loss rates were generally close to the TLS-measured values, and a linear function could be used to describe the relationship between the predicted and measured soil losses. The model efficiency of 0.86 illustrated the good fitness of the predicted soil loss rates after revision by the functions shown in Table 3.

### 4.3. Implication and Limitations of This Study

The results of this study showed that the WEPP hillslope version could provide reasonable predictions of soil loss from short gentle roads in the red soil region of China. It also illustrated that the model inputs, including soil parameters such as rill erodibility and hydraulic conductivity, suggested by the WEPP:Road were acceptable for unpaved roads in the study area. Therefore, the WEPP hillslope version could be used to calculate the total soil loss amount from unpaved road surfaces in the case of road lengths less than 100 m and with relatively low gradients. Meanwhile, the WEPP simulation could fit the detailed soil loss and the peak erosion in long road segments despite the underestimation. This result implies that the model could predict high soil loss risk points along roads. This information would be helpful for road conservation and erosion control. The equations developed in Table 3 could be used to improve the precision of the WEPP simulation. Since the WEPP model and the revising equations can accurately predict road surface soil loss and can tell where the peak erosion will occur along roads, we can conclude that the WEPP is a suitable for road erosion modeling in the study area. To further enhance the WEPP efficiency for detailed road erosion modelling, the intercepted upstream area and road tortuosity should also be considered in future studies.

It should also be noted that there are limitations in this study. Since the measured soil loss amount is essentially rill erosion, which is calculated based on one-time TLS scanning and a referenced road surface [4], neither the interrill erosion nor the sediment deposition can be precisely measured. Although interrill erosion composed a very small percent of most roads with high soil loss in this study, it might contribute to the deviation of simulation compared with the measured soil losses along some short and gentle road segments. Further multiperiod dynamic TLS-aided erosion measurements should be conducted to provide more reasonable criteria. Meanwhile, the soil inputs, including rill erodibility, critical shear stress and effective hydraulic conductivity, used in this study were the same for all road segments. Considering the variation in soil properties throughout the watershed, the detailed road surface soil parameters should be quantified for different road segments or along roads. The small watershed scale might be another limitation of the results. More types of unpaved road networks which composed of different design, traffic level, soils and terrain conditions should be selected to enhance the representativity and reliability.

## 5. Conclusions

This study validated the performance of the WEPP hillslope version in predicting unpaved road soil loss in the red soil region of South China. The results illustrated that the WEPP-simulated soil loss generally provided reliable estimation for road segments shorter than 100 m and with gradients lower than 10%. In the case of road lengths exceeding 100 m or gradients exceeding 10%, the WEPP simulation would underestimate the total road surface soil loss. The detailed prediction of soil loss along roads revealed that underestimation mainly occurred in the case of the peak erosion segments, which could be attributed to the linear critical shear stress model that cannot fit the nonlinear mechanism of rill detachment in road surfaces. Meanwhile, the underestimations of WEPP output along roads likely increase with slope length but decline with slope gradient, which might be explained by the sediment equilibrium theory. Despite the underestimation, the WEPP-simulated soil losses showed a close relationship and were consistent with the trend of measured values along road segments. The maximum soil loss positions could be reasonably predicted by the WEPP model. The lack of upstream flow area might also lead to the underestimation of steep and long road segments in this study. An equation of upstream flow area per road length could be established to estimate the model deviation and enhance the WEPP performance. The above results are helpful in evaluating unpaved road soil erosion and expanding the range of WEPP applications.

## Figures and Tables

**Table 1:** Terrain factors and the TLS-measured erosion for each road segment.

CODE	Length (m)	Slope (%)	RT *	UA * (m^2^/m)	Erosion (t/a)	CODE	Length (m)	Slope (%)	RT *	UA * (m^2^/m)	Erosion (t/a)
R1	340.0	13.9	1.43	34.41	58.49	R11	91.3	1.5	1.50	13.14	0.14
R2	45.2	10.4	1.03	6.64	3.93	R12	89.0	5.0	1.37	22.47	0.53
R3	72.4	6.3	1.11	19.34	8.96	R13	85.1	3.8	2.14	11.75	0.64
R4	208.5	14.8	1.21	35.97	17.93	R14	159.3	9.3	1.27	7.53	2.57
R5	103.4	17.4	1.14	117.02	21.31	R15	67.9	4.0	1.07	19.15	0.23
R6	195.0	9.3	1.41	46.67	44.65	R16	389.5	12.9	1.67	28.50	21.25
R7	101.1	8.3	1.17	44.51	7.98	R17	73.8	6.5	1.03	16.26	0.15
R8	300.5	10.7	1.26	35.27	12.15	R18	50.3	3.5	1.12	11.93	0.08
R9	213.3	9.0	1.18	30.47	48.02	R19	251.1	10.5	1.97	9.16	11.90
R10	106.4	6.9	1.27	16.92	0.19	R20	271.4	12.3	1.21	25.06	38.82

* RT represents the road tortuosity which was calculated as the total road segment length divided by the straight length between two ending points of a road segment. UA represents the intercepted upstream drainage area per road length; the intercepted upstream drainage area was measured by GIS based hydrological analysis [4].

**Table 2:** Soil parameters for road surface erosion modelling with WEPP.

Input Soil Parameters	Values
Albedo of the bare dry surface soil	0.6
Initial saturation level of the soil profile porosity (m/m)	0.5
Baseline interrill erodibility parameter (kg·s/m^4^)	500,000
Baseline rill erodibility parameter (s/m)	0.0001
Baseline critical shear parameter (N/m^2^)	2
Effective hydraulic conductivity of surface soil (mm/h)	3.8
Depth from soil surface to bottom of soil layer (mm)	200

**Table 3:** TLS-measured soil loss as a function of WEPP-predicted soil loss along roads.

CODE	Equation	R^2^	SIG	N	CODE	Equation	R^2^	SIG	N
R1	Y = 1.555x − 1.044	0.507	0.000	67	R11	Y = 0.892x − 0.182	0.523	0.001	18
R2	Y = 0.042x^3.824^	0.926	0.000	9	R12	Y = 0.468x + 0.086	0.506	0.006	13
R3	Y = 0.297x^2.665^	0.782	0.000	13	R13	Y = 0.797x + 0.526	0.053	0.472	12
R4	Y = 0.955x^0.856^	0.283	0.000	40	R14	Y = 0.441x + 0.991	0.514	0.000	25
R5	Y = 0.270x + 16.44	0.008	0.712	20	R15	Y = 0.722x − 0.666	0.746	0.006	8
R6	Y = 6.934x − 41.49	0.278	0.039	36	R16	Y = 0.450x^1.015^	0.645	0.000	76
R7	Y = 2.657x + 3.68	0.312	0.013	19	R17	Y = 3.022 − 0.295x	0.106	0.675	4
R8	Y = 0.867x^0.805^	0.419	0.000	58	R18	Y = 2.095x − 1.450	0.688	0.041	6
R9	Y = 7.319x − 42.51	0.591	0.000	42	R19	Y = 1.098x − 3.369	0.068	0.131	35
R10	Y = 1.46 − 0.140x	0.125	0.235	13	R20	Y = 1.841x − 1.257	0.202	0.001	51

**Table 4:** The correlation coefficient between model deviation and influential factors.

	Length	Slope	Road Tortuosity	Upstream Area	Unit-UA *
Pearson R	0.034	−0.331	0.304	−0.340	−0.506 *
SIG	0.886	0.154	0.193	0.142	0.023
N	20	20	20	20	20

* Unit-UA stands for upstream flow area per road length.

**Table 5:** The correlation coefficients between model deviation along road and influential factors.

Code	Distance		Slope		N	Code	Distance		Slope		N
	Pearson R	SIG	Pearson R	SIG			Pearson R	SIG	Pearson R	SIG	
R1	−0.015	0.906	−0.322 **	0.008	67	R11	0.325	0.189	−0.073	0.780	18
R2	−0.761 *	0.017	0.003	0.995	9	R12	−0.354	0.235	0.373	0.233	13
R3	−0.705 **	0.007	−0.173	0.590	13	R13	−0.653 *	0.021	0.243	0.447	12
R4	0.144	0.374	−0.204	0.213	40	R14	−0.331	0.099	0.689 **	0.000	26
R5	0.277	0.224	0.019	0.937	21	R15	0.118	0.780	0.475	0.234	8
R6	−0.466 **	0.004	0.076	0.660	36	R16	0.451 **	0.000	0.035	0.763	76
R7	−0.025	0.919	−0.290	0.243	19	R17	−0.617	0.383	0.475	0.525	4
R8	−0.119	0.370	0.369 **	0.004	59	R18	−0.499	0.314	0.632	0.178	6
R9	−0.426 **	0.005	−0.368 *	0.016	42	R19	0.220	0.204	0.053	0.761	35
R10	−0.864 **	0.000	0.843 **	0.000	13	R20	0.034	0.815	−0.232	0.109	50

* and ** respresent the significance level of α = 0.05 and 0.01 respectively.

## Data Availability

The data that support the findings of this study are available from the corresponding author upon reasonable request.
